# Decoding the Psychiatric Space: Cross Country Comparison of Facilities for Mental Health Service Users

**DOI:** 10.3390/ijerph19148832

**Published:** 2022-07-20

**Authors:** Evangelia Chrysikou, Eleftheria Savvopoulou, Jane Biddulph, Gabrielle Jenkin

**Affiliations:** 1 The Bartlett School of Sustainable Construction, University College London, London WC1E 6BT, UK; 2 Independent Researcher, 10676 Athens, Greece; syn-thesis@hotmail.com; 3 Institute of Epidemiology and Health Care, University College London, London WC1E 6BT, UK; jane.biddulph@ucl.ac.uk; 4 Department of Psychological Medicine, University of Otago, Wellington 6242, New Zealand; gabrielle.jenkin@otago.ac.nz

**Keywords:** mental health, mental health facilities, psychiatric architecture, de-institutionalisation, normalisation theory

## Abstract

Normalisation theory made perfect sense at the onset of de-institutionalisation. To map its influence on mental health facilities, research was conducted and began with ten facilities within England (UK) and France, followed by a further two in England and four in New Zealand. A checklist tailored to mental health facilities was used to measure the extent to which the facility looked domestic or institutional. Hence, the mental health checklist architecturally measured domesticity versus institutionalisation in psychiatric architecture. It consisted of 212 features, grouped into three main categories—context and site; building; and space and room—and was based on a pre-existing checklist designed for hostels for those with learning disabilities. The mental health checklist was developed and piloted in Europe and reflected European de-institutionalisation principles. Cross-country comparison revealed that patient acuity was potentially not a determinant of institutional buildings for mental health. Institutional facilities in France were detected, and some of the most domestic facilities were within England, with the most recent sample having a greater tendency towards the more institutional end. Those in New Zealand tended towards the most institutional. Across all 16 facilities, there were very few universal institutional and domestic features, raising the ambiguity of a clearly defined stereotype of facilities for mental health service users. Consequently, the current fluidity of design across and within countries provides a significant opportunity for designers and mental health providers to consider non-institutional design, particularly at the planning stage. The use of the mental health checklist facilitates this debate. Future research in other geographical areas and through further consideration of cultural differences provides further opportunities to extend research in this area, with the potential to enhance and improve the lived experience of users of mental health services.

## 1. Introduction

In the 1990s, with progress towards care in the community as an alternative to care within psychiatric hospitals and the psychiatric ward of the general hospital, there was a degree of ambiguity about what the new model should look like. Neither the campus-like structure of the psychiatric hospital or the ward, which had been essentially a replica of a typical hospital ward and defined by the functions of inpatient surgery and pathology, were fit for purpose; the new psychiatric structures in the community should be small-scale, spread across the catchment area and locally defined, in terms of function, brief and typology. This led to an extensive variety in facility provision and the opportunity for experimentation. Parallels with the neighbouring field of learning disabilities emerged as normalisation theory [1] appeared to influence community mental health in general and in particular in the design of its premises. Normalisation theory appeared compatible with the psychosocial rehabilitation movement of mental healthcare and the idea of valorisation, whereby patients relearnt skills, routines and practices that were missing within the asylums, such as eating with cutlery, having personal belongings or clothes, and eventually—at the end of their social reintegration—having a place to call home, even if that might be a protected apartment [2]. The emphasis of the normalisation theory on domesticity made perfect sense at the time of de-institutionalisation efforts across Europe.

However, in practice, (a) domesticity in architectural terms was never defined, especially for psychiatric environments and at that time remained outside the scope of architectural research; and (b) 24 hstaffed acute wards, even in the shape and form of an 8–12 person household, were still part of the healthcare system. Their physical context was still that of healthcare buildings, and healthcare buildings have been considered to reflect the prevailing socio-political system [3]. As a home is connected with a larger degree of autonomy than that of an institution, that level of autonomy could not be applicable in a dwelling where residents were sectioned and staff were the visitors who had significant powers over the residents. However, the mental health facilities may still remain closer to the institutional paradigm that they were originally set to replace and have been described as small institutions in the community [4].

To understand if normalisation theory could constitute a realistic model for psychiatric wards, a series of tools were created or adapted by the author, EC, from pre-existing tools that were applied to other contexts. A mental health checklist that aimed to measure a psychiatric facility for domestic or institutional traits was developed by the author EC. One of the purposes of the mental health checklist was to enable the evaluation of significant design building features of mental health facilities, as a tool that architects/designers could easily consult during the design process to ensure they could avoid unnecessary institutional solutions. The opportunities for non-institutional solutions in the design of buildings has the potential to enhance and improve the lived experience of mental health service users and yet is infrequently considered. The checklist could also act as a scale that enables comparison across psychiatric facilities, in terms of domestic versus institutional features and therefore acts as a scale to architecturally measure domesticity versus institutionalisation in psychiatric architecture [5].

The aim of this study was to compare mental health facilities from different countries (England, France and New Zealand), in terms of domestic or institutional traits through the use of the mental health checklist and gain further insight into institutional undercurrents of domestic psychiatric built environments.

## 2. Materials and Methods

### 2.1. Development of the Mental Health Checklist and Piloting

The mental health checklist was developed by the author EC in the late 1990s and inspired by an earlier checklist by Robinson et al., 1984, which was specific to hostels (accommodation) for those with severe learning disabilities [6]. The adaptation of this checklist primarily removed those aspects that were specific to learning disabilities and those features that were not applicable to the mental healthcare context; for example, in healthcare premises, drug cabinets would not exist within bathrooms as drug storage was within the clinics. The mental health checklist also included aspects that would be potentially relevant in a health setting and based on a wide range of site visits; for example, the integration and placement of a toilet inside the patient bedroom was not unusual in the French healthcare sector. The development of the checklist drew from facility knowledge gained from visits by EC to 400 mental health facilities across Europe including Greece, Belgium, France and the UK. This included visiting 200 facilities across the UK and 40 in France, and facilities from these geographical areas were included within this study.

The Robinson et al. checklist [6] was designed for a defined building type, which was essentially a hostel for adolescents with learning disabilities and focussed on a North American setting. In contrast, the mental health checklist was designed for the mental health ward, which was a clearly defined healthcare concept—a ward catering for patients at the acute spectrum of care—and not an architectural concept. Mental health wards could be housed in different building types ranging from a house to the hospital ward typology, with several options in between.

The checklist by Robinson et al. had employed normalisation theory for learning disabilities [6], which had been clearly defined as a concept, and the premises were residential. However, normalisation in psychiatric care was not directly translated, and the premises were never meant to be homes as they were part of the psychiatric healthcare provision. Therefore, the theory could not be directly applied, but the basis was that facilities should look homelike but in the healthcare context.

The concept of domesticity within the mental health checklist was referring to homelike features for healthcare premises rather than purely residential, and therefore it should be viewed as enabling a more critical approach to domesticity and normalisation, especially as clinical premises were also included, such as the presence of a clinic, a seclusion room or an electroconvulsive therapy facility. This viewpoint also facilitates the adaptability of the mental health checklist to other healthcare premises, not necessarily accommodation, whilst examined under the prism of normalisation; for example, its applicability to drug treatment facilities through a further adaptation of the mental health checklist undertaken by Dr Suzanne Spear from the Department of Health Sciences, California State University [7].

Further modifications were that the Robinson et al. checklist did not include a non-applicable option and was visually descriptive including detailed descriptions and images [6]. However, the mental health checklist had to be applicable to diverse typologies and contexts that made stereotypical representation challenging and potentially misleading. Therefore, the checklist was primarily adapted to reflect the functions of a community psychiatric ward and at the same time underwent extensive simplification in order to become less context specific.

The Robinson et al. checklist is comprised of a total of 236 features versus 212 in the mental health checklist. The mental health checklist consists of features relevant to the building, the building’s setting and its placemaking and grouped into three main categories of context and site (22 features), building (40 features) and space and room (150 features) [5]. Each feature is rated as either institutional or domestic. Elements that were similar to what could be seen or what existed in the nearby housing were considered to be domestic. Institutional elements could be elements that were associated with public building architecture. If a feature was not relevant, the researcher could classify it as not applicable.

In 2000, a pilot case study of St Pancras hospital (London, UK) was used to assess the checklist and adjust the checklist prior to its use. Data from the pilot study were not included in the facility series for England within this study.

### 2.2. Mental Health Facility Selection and Data Collection

Exemplary examples of facilities might be considered supportive for proof of concept research. A series of case studies occurred from 2000 onwards. Data from 16 facilities across three countries were included in the current study: England (*n* = 7), France (*n* = 5) and New Zealand (*n* = 4). Data were collected from five acute mental health wards in England and five in France in 2000–2004, two further acute mental health wards in England in 2015–2017 and four mental health facilities in New Zealand in 2017–2020.

In England and France, the checklist was completed by the author EC and in New Zealand by GJ, with oversight from E.C. This ensured scoring consistency across the entire series. The mental health checklist was designed by an architect (EC), but the application by GJ, a social scientist, provided further opportunity to consider the possibility of use by other disciplines. Cultural differences existed between Europe and New Zealand; for example, cultural norms for shared or single accommodation potentially existed. Within the New Zealand series, for some of the checklist items, there were instances where both institutional (I) and domestic (residential, R) features appeared and were, on consideration, classified as institutional. This applied to one facility regarding the bedroom floor material selection, as all bedrooms did not share the same flooring; one facility regarding seating arrangement in the lounge, as more than one arrangement existed and thirdly; and for two facilities where not all bedroom doors with watch panels within the facility had curtains on the inside.

#### 2.2.1. Selection of Facilities within England, UK

The five facilities from the 2000–2004 series were located in Middlesex (South-East England), Sheffield (North-Central England), Hull (North-Eastern England), Birmingham (West Midlands) and St Albans (Hertfordshire). These were selected based on their innovative planning and design. Four out of five were included in the 1997 Health Building Note 35 “Accommodation for people with mental illness. Part 3: Case studies” [8]. Both facilities from the 2015–2017 series were located in London and representative of the facilities within this geographical area. One of these was considered dated and obsolete at the time of the earlier series and was about to be demolished. However, it remained operational and so was investigated during the later time period. The other facility was within a former hospital converted into a psychiatric campus, which was completely remodelled in 2004 to accommodate community mental health.

#### 2.2.2. Selection of Facilities within France

The five facilities in France were located in Paris (two facilities), Pas de Callais (Northern France), Mouans Sartoux (South France) and Cote d’Azur. They were selected from a smaller pool of available facilities. The main criterion was to match the facility patient population to that in the series in England. As France had retained their psychiatric hospitals, some of the patients who were comparable with England would be in psychiatric hospitals at that time. Consequently, French community mental health provision was potentially further from the more acute end of the spectrum. The five facilities were selected from premises within the community but as close to the acute spectrum as possible.

#### 2.2.3. Selection of Facilities within New Zealand

The New Zealand facilities were selected using a diverse case selection method from a pool of 20 [9]. Diversity was maximised using building age, condition and location as criteria. Therefore, the series included a selection of old and new buildings, whether attached to a hospital or on their own campus, and in small and large cities around the country.

### 2.3. Analyses of Domestic and Institutional Features within, between and across All Countries

For each facility, each mental health checklist feature was classified as either Institutional (I), Domestic (Residential) (R) or Not applicable. Using data combined from all facilities (*n* = 16), for each checklist feature, excluding those features that were not applicable to all facilities, the frequency classified as either I or R was calculated (not all data have been shown). Of the 212 features within the mental health checklist, a total of 27 features were not applicable to one or more of the facilities—for context and site features, 0/22 were excluded; for building features, 5/40 were excluded; and for space and room features, 22/150 were excluded from the all-facility comparison. However, the total frequency of institutional features by each of the three checklist categories (context and site, building and space and room) by country and by facility have been derived both with and without the inclusion of non-applicable features for comparison.

Common features within facilities within each country, comparing England versus France, England versus New Zealand, France versus New Zealand and those that were common across all countries were identified. Potential temporal comparisons were also investigated through comparison of facilities in England from each time period of investigation—2000–2004 and 2015–2017—and all country facilities across the two time periods of 2000–2004 and 2015 onwards.

## 3. Results

### 3.1. Domestic and Institional Features within Facilities in England, France and New Zealand Combined (n = 16)

#### 3.1.1. Context and Site Features

The majority of facilities were located in residential areas (63%: R), but only 38% (R) had immediate neighbours that included housing (Table 1). Most had shops within the neighbourhood (88%: R), public parks or recreation areas that were within walking distance (81%: R) and had paved pedestrian paths (75%: R). For the majority, the building façade was similar to adjacent buildings and also had windows of different sizes according to function (63%: R). The use of building materials such as stucco, brick and stone also helped to blend the facility within the community, and these were used in 12 cases (75%: R). However, most were differentiated in size from adjacent buildings; differed in distance from the street compared with the neighbourhood, which made them appear more institutional; and had different unit parking than at the adjacent buildings, in relation to, size, location and availability. Furthermore, almost half had different colours and material selections from nearby buildings (44%: I), which made them stand out to a greater extent. The more institutional face of the facilities was also reinforced in 75% (I) of facilities through displaying a label or inscription by the entrance, and 63% (I) did not have a designated waiting area outside the entrance.

#### 3.1.2. Building Features

The majority of facilities (81%) had institutional features of more than one door at the entry and were of a size similar to public buildings (75%). Most facilities had a door that was open to the public (69%: I), and all had a door that opened out or automatically (I). Two facilities had a sliding or revolving front door (I). Most had a security desk or reception area at the entry point (69%: I), and almost all did not have a coat hanging area near the entrance (94%: I).

Almost half had a living room area just by the ward/facility entry (44%: R), and the majority had social spaces; for example, a kitchen and dining area that were grouped together (63%: R). In terms of decoration, 44% used wall decorations, pictures or wallpaper at the entrance or within circulation areas, and 44% used decorative lighting fixtures within these areas (R). The residential feature of the presence of an individual mailbox for users that could be directly accessed by the post service was absent in all facilities. 

Within the circulation areas, institutional features within the majority of facilities included long corridors within the autonomous wards/areas (88%); 75% had a corridor width of more than 1.20 m; all had notices on the walls or doors; 94% had exit signs on the walls; and 19% had at least one mirror at a corner to allow visibility of a corridor turning point or room. Enlarged corridors that created sitting areas without any windows to the outside (I) were present in 44% of facilities. Predominantly wood, cork, tiles, marble, mosaic or carpet were used in 75% of facilities, as opposed to the institutional feature of linoleum, vinyl or concrete, and only 38% chose the institutional feature of fluorescent lighting within these areas.

The majority of facilities had direct access to the outdoor areas from social spaces, such as the kitchen, dining or lounge area (75%: R), and almost all had an outside space that was visible from the interior social areas (94%: R). However, access to the outdoors was locked or partially locked in 56% (I) of facilities. Only one facility had the institutional feature of a bathroom opening directly onto a social space rather than a corridor or bedroom, and the majority had bedroom interiors that opened onto a public corridor or area (56%: I).

#### 3.1.3. Space and Room Features

##### General Features

Most facilities possessed specialised activity rooms, such as for games, music, arts and crafts (69%: I), and 19% (I) had internal spaces that were used by external users only. Two had social areas, such as a dining or kitchen for external people where users could not be present (I). Institutionalisation was strengthened through the presence of an administration office in the ward (94%) and the presence of more than one office (81%). Additionally, in most cases, there was a dedicated staff room in the ward (81%), but only 19% had a dedicated staff room for sleep. Almost all had automatic door closers (88%: I), and in the majority, some doors had crash bars or kick-plates (56%: I).

All had a psychiatric office attached (I), and 69% had a psychiatric consultation/interview room (I). Only one facility, which was in France, had a GP (general practitioner) office attached. Four had a seclusion room (I), and for 69%, an electroconvulsive therapy facility was attached (I). All facilities had a clinic within the ward (I).

##### Common Areas—General Features

In most cases, there were more than six residents in an autonomous area (81%: I). The residential feature of two or three social areas was present in 50% of facilities, and different colour or decoration was utilised (50%: R). The majority had a different furniture selection for each area (69%: R). Within the lounge area, the majority of facilities did not have a fireplace (88%: I) but had a television (88%: R).

Most had comfortable seating, such as a sofa (88%: R), but half had seating arrangements such as seating placed against the walls (I). A coffee table in front of a sofa or armchair was present in 50% (R).

The majority of facilities incorporated a variety of decorative objects, vases or plants (56%: R), had pictures on the as walls (63%: R) and had shelves or side tables with objects and/or books in the lounge (63%: R). Less than half had the institutional features of signs or notices on the lounge walls (44%), and only 31% had stands with leaflets. Most had external views from the lounge (88%: R), with 44% having a window or door to the outside that could be fully opened by users unsupervised (R). Most had a lounge ceiling with sprinklers and smoke detectors (75%: I). However, the majority had floor material such as carpet, marble, ceramic tile, wood or cork (75%: R), and only 38% used overhead fluorescent lighting only (I). Most of the living rooms had windows with curtains or shades (69%: R), and only 19% had a TV room (I) instead of a lounge with a television.

##### Dining Room Features

Most facilities had a dining area located within the facility rather than in another building or unit (88%: R) and located relatively close to the lounge (69%: R). In a minority of facilities, there was a separate dining room for staff (25%: I). The majority had institutional features that included more than one table in the dining room and smoke detectors, sprinklers or/and alarms in the dining area.

##### Kitchen and Kitchenette Area Features

Most had more than one kitchen (63%: I), and 50% had a kitchen for professional use and a different kitchenette for service users (I). Users had access to a domestic cooker and a fridge, and food could be prepared in 31% of facilities (R). In 56%, a microwave was present (R), and tea or coffee could be prepared in the kitchen area (75%: R). However, food came in frozen and was reheated in a trolley in 31% (I) of facilities and was served through a designated passthrough in 38% (I). A vending machine or water cooler was present in 44% (I), and locked cupboards—for example, for food or cleaning products—were present in 81% (I).

##### Laundry Area Features

In most cases, there was a laundry service (75%: I), but laundry facilities that were near the kitchen or the bedroom area were provided in 69% (R). Almost all facilities had locked storage areas (94%: I).

##### Bedroom Area Features

Bedrooms were shared in 44% (I) of facilities, but only 25% (I) had more than two people sharing a room. Most windows had restricted opening (75%: I), and 19% (I) had bars. In most, the bedrooms were not identical in shape, window location and window size (56%: R).

Within the majority of facilities, the bedroom doors opened inwards (56%: R), and service users could lock the door from the inside in 50% (R) of facilities, and from the outside in 25% (R). Within most, the bedroom doors had name tags, numbers or decorations on them (81%: I), and 56% (I) had watch panels, and only two with watch panels had curtains on the inside.

Most facilities had bedrooms with identical furniture (88%: I), and 44% (I) had a hospital bed in the room. In 69% (I), there was a washbasin, and 19% (I) had a toilet. Only 13% (I) had a track of privacy curtain from the bedroom ceiling.

An individual desk for each service user was available in 63% (R), an individual double wardrobe was found in 50% (R), 56% (R) had a side table for each service user, and 31% (R) had an individual chest of drawers (R). In 50% (R), a bookshelf was present in each bedroom, and 63% (R) had an armchair for each service user in the bedroom. Carpet or rugs was present in 38% (R), and the majority had stereos/radios or a television set in the bedroom (69%: R). There were posters, decorations or pictures in 63% (R), but in 31% (I), there was a framed area on the wall for pictures. Half of the facilities had at least some bedroom furniture fixed (50%: I), and 19% (I) had all of the furniture fixed. In most cases, the mirror in room was plastic (63%: I), and over half of the facilities had curtains, colours and bedspreads in the bedrooms that were the same colour and style in all bedrooms (56%: I). Most had curtains for the windows (69%: R).

Fluorescent lighting was used in 56% (I) rather than a variety of lighting, such as adjustable or bedside lighting, and 75% (I) had sprinklers and alarms fitted. However, plastered ceiling instead of suspended tiles was used in the majority of bedrooms (88%: R).

In most facilities, bathrooms were located in the bedroom area (75%: R), and service users did not have to pass through public areas to access them (75%: R). En-suites were available for some rooms in 63% (R) of facilities, with only 25% (R) having it for all rooms. In 69% (R), the bathroom had a bathtub or a bathtub and shower for service users. Half of the facilities had a shelf in the bathroom (R), one sink (94%: R), one toilet (94%: R) and one shower (81%: R), with 38% (R) of facilities having curtains around the shower. Most bathrooms did not have a hoist (94%: R). Institutional features included handles by the toilet (63%), wheelchair accessibility in some or all bathrooms (63%), hospital bins in the bathrooms (50%), a fixed soap dispenser (56%) and a paper towel dispenser or hand dryer (50%). None of the bathrooms had urinals (0%: I).

Less than half of the facilities had decorative elements in the bathrooms (31%: R), and all but one had a bathroom window.

##### WC in Common Areas Features

In 63% (I) of facilities, there was a staff WC in the wards. In common areas, the majority had one toilet (81%: R), a paper towel dispenser (81%: I), fixed soap dispenser (75%: I) and a hospital bin (56%: I). Only one facility had an automatically self-cleaning toilet seat (I).

### 3.2. Features by Country (England, France, New Zealand)

Table 2 displays the institutional feature score for each facility by each of the categories from the mental health checklist—context and site, building and space and room. Facilities within New Zealand had the highest percentage of institutional features, with an average of 64% (range 59–68%), followed by England with 47% (range 23–61%) and France with 42% (28–49%). France, on average, consistently had a lower institutional score than England or New Zealand in each of the three categories—context and site, building, space and room—and New Zealand, on average, consistently scored higher than the other two countries.

Comparing facilities in England by time period—2000–2004 (facilities 1–5) versus 2015–2017 (facilities 6–7)—those from the more recent time period had a greater proportion of institutional features overall; 57% (212/370) versus 42% (393/925). This difference predominantly arose from an increased prevalence of institutional features within the category of space and room, at 53% (136/256) versus 38% (243/640). Across all categories of the checklist, the facilities from 2015–2017 had a higher frequency of institutional features. The increase in frequency of institutional features within the category of building showed the least percentage rise within the most recent time period, at 66% (46/70) versus 54% (94/175).

When the frequency of institutional features from facilities from the time period 2000–2004 (England facilities 1–5 and within France) were compared with those from 2015 onwards (England facilities 6–7 and within New Zealand), those from the most recent time period had a comparatively higher frequency across all of the checklist categories:context and site—70% (93/132) in 2015 onwards versus 45% (99/220); building—71% (150/210) versus 54% (189/350); space and room—58% (444/768) versus 38% (492/1280); and overall—62% (687/1110) versus 42% (780/1850).

#### 3.2.1. Common Features of Facilities in England

Within England, there were 6 common characteristics for context and site features, of which 4 were institutional; 7 common building features, of which 4 were institutional; and 29 common space and room features, of which 11 were institutional.

In terms of context and site and building features, across the facilities, none had immediate neighbours including housing; they were differentiated from their neighbours by their parking area, their front garden and fence selection and their location, in terms of distance from adjacent buildings. Shops, parks or recreation areas were within walking distance from the facility. Their front door opened out or automatically, and they did not have individual mailboxes for service users. There were exit signs and notices on the circulation areas’ walls.

Common institutional space and room features included the inclusion within the facility of psychiatric offices and a clinic, the existence of locked cupboards and storage areas, the absence of both diversity in the bedroom design and a mirror in the bedrooms. Users were unable to lock their bedroom door from the outside, sprinklers and alarms were present on the ceilings, and there were restrictions in window opening.

#### 3.2.2. Common Features of Facilities in France

The were no common institutional characteristics for building context and site. There were two common residential features for context and site. Facilities were all located in the immediate vicinity to neighbours including housing, and their primary building materials were stucco, brick and stone. They shared 13 common building features, of which 7 were institutional. Institutional features included having a front door opening out or automatically, not having a coat hanging area or individual service user mailboxes and having long corridors with exit signs and notices on their walls as well as an administration office at the entrance.

There were 28 common space and room features. The majority were domestic features, with only seven being institutional and included having staff offices (administration, psychiatric offices, staff retreat space) within the facility, locked storage areas and closets. It is interesting that of the common features, facilities in France had a higher percentage of residential common features than facilities in either England or New Zealand and were related to space design and quality including social spaces, bedrooms and bathrooms.

#### 3.2.3. Common Features of Facilities in New Zealand

Across the four facilities, they had eight common characteristics in terms of context and site features. All of these features were institutional, and included a building exterior that stood out from the adjacent buildings due to a different build size, distance from the street and between the facility and the adjacent buildings and a different front width relative to nearby houses. The access point to the facilities differed from nearby houses, the unit parking stood out (in size, shape or location) in comparison with nearby buildings, and the front garden had different fencing and landscaping to the adjacent ones. Furthermore, there was a label or inscription before or at the entrance.

There were 20 common building features, of which 18 were institutional. Specifically, the front façade of the buildings differentiated from adjacent ones by having more than one front entrance door similar in size to public buildings and from different material than adjacent residential ones, and the doors opened automatically and were open to the public. There was a security desk/administration office at the entrance and no coat hanging area, although one had lockers for visitors. The facility entry was connected to a lobby or waiting area without being differentiated from the circulation area. There were no individual mailboxes for service users or decorative lighting features. The lighting in the facilities was mostly artificial (fluorescent) and fixed. There were notice and exit signs on the walls. There was direct access to outdoor areas from social spaces as all users had to have access to courtyards (usually during 9 a.m. to evening). These areas were sometimes locked/partially locked at night.

There were 67 common room and space features, and 41 were institutional. For example, common features included specialised activity rooms equipped to accommodate these activities, such as arts and crafts or music. Apart from the administration office, there were other staff rooms such as offices, a dedicated room for staff to retreat, multidisciplinary offices, a consultation/meeting room, clinic and a separate ward/wing from the “open” ward for the seclusion room. Common institutional characteristics within social spaces included the use of the same decoration and colour selection across social spaces. They did not have features such as a fireplace, decorative objects or plants. There was less privacy for service users as there were more than six users in an autonomous area. Lighting was again fluorescent and overhead only. The ceiling had sprinklers and smoke detectors. The dining area had more than one table for eating. Within the kitchen, there was a separate area for preparing the food and washing up; service users did not have access to a domestic cooker/fridge to prepare food there, and facilities had more than one kitchen. A laundry facility was also provided in the facility for service users to wash their own clothes as sheets were washed in the main hospital. There were external views from social spaces, but windows were mostly restricted from opening. Some residential features within the social spaces included the selection of furniture (having different and comfortable sitting spaces) and the inclusion of a television. The kitchen equipment was not commercial, and service users were able to prepare tea or coffee if they wanted to. The bedrooms had institutional characteristics by being identical in shape and window size and there was a watch panel at the door of the room. Some rooms had fixed furniture, and there were no decorations/posters on the walls, stereos, television set, armchairs, rugs or carpets and mirrors. Some bedrooms had en-suite bathrooms. Lastly, all bathrooms had hospital bins, handles by the toilet, a fixed soap dispenser, a paper towel dispenser/hand dryer (the same as those for the WC in common areas), one toilet and one shower and did not have decorative elements, urinals or hoists.

### 3.3. Cross-Country Comparison: Common Features between Countries

The features from the mental health checklist that were common across geographical regions were identified. Comparing the facilities in England with France, nine common features were identified; see Table 3. Comparing facilities in England with those of New Zealand, 18 features common features were identified, and 15 were identified when comparing France and New Zealand. Six features were common to all countries.

#### 3.3.1. Cross-Country Comparison: Common Features England versus France

There were nine common features, and six were institutional ones. The facilities did not share any context and site features. They shared three institutional building features: all had front doors that opened either automatically or opened out, did not have individual mailing boxes for service users, and they all had notices on circulation areas walls or doors. The other three institutional features concerned space and room and included a clinic in the ward and a psychiatric office that was attached or included, and locked storage areas and closets. The common space and room residential features included the absence of a seclusion room within the facility and that their bathrooms did not have urinals.

#### 3.3.2. Cross-Country Comparison: Common Features England versus New Zealand

Facilities in England shared more common features with those in New Zealand compared with those in France. They had 18 common features, of which eight were institutional. Of the institutional features, three were common context and site features, three were building features, and two were space and room features. Of the common context and site features, all common features were institutional, including the use of a different distance between the facility and the adjacent buildings and differences in unit parking and the front garden. All common building features were also all institutional; their front door opened automatically or opened out, they did not have individual mailboxes for service users, and they had notices on circulation walls and doors. There were 10 common space and room features. The common institutional features included psychiatric offices that were attached or in the facility, as well as a clinic. The common residential features were that they did not have a GP office, there was a television in the lounge area, no walk-in cooler, bedroom bathrooms had only one sink, one toilet, one shower and no urinals, and the WC in common areas did not have automatically self-cleaned toilet seats.

#### 3.3.3. Cross-Country Comparison: Common Features France versus New Zealand

France and New Zealand mostly shared institutional features, and these comprised 12 of the 15 shared characteristics. Specifically, they shared 7 features in terms of building features, and all were institutional. These included a front door that opened out or automatically, and there was no closet or area at the entrance for hanging coats or individual mailboxes directly accessed by post service (see Table 3). There were 8 shared space and room features, of which only 3 were residential features.

#### 3.3.4. Cross-Country Comparison: Common Features across All Countries

By comparing the features from the mental health checklist across all facilities, there were only six common characteristics that appeared in every facility irrespective of country. Five of the common features were institutional: having notices on circulation areas’ walls, psychiatric offices and a clinic inside the ward, having a front door that opened automatically or opened out and the absence of individual mailboxes directly accessed by the post office. The single common residential feature was that there were no urinals within bathrooms. They did not share any common features in terms of location and site features.

## 4. Conclusions

An investigation and comparison of institutional and domestic (residential) features of community mental health accommodation building stock across three countries, within and between countries was conducted using a mental health checklist. In all three countries, we examined the settings that were available within the community at the most acute end of the spectrum for each country. Due to systemic differences, and mostly in relation to psychiatric hospital care within each country at the time, some variation in patient acuity in community care between countries may exist. The facilities in England and New Zealand have a potentially comparable population of mental health service users, but the patient group within community care in France was potentially further from the more acute end of the spectrum as France had retained its hospitals. The mental health checklist revealed that acuity was potentially not a determinant of institutional versus domestic buildings for mental health, as we could detect fairly institutional facilities in France but also some of the most domestic cases in England. The most recent examples of facilities in England included within the series tended to be closer to the institutional end and as a result of increased anti-ligature. Those in New Zealand tended towards the most institutional end of the entire series. This may indicate that although de-institutionalisation has been actively promoted by policy, including the World Health Organisation [10], building stock may have tended to become more institutional over time.

The mental health checklist presents four scores for each country and overall average scores based on a diverse range of facilities within countries. New Zealand appeared the most institutional in terms of all features. A spatial analysis using architectural morphology tools might be able to indicate if NZ layouts promote social interaction. A spatial analysis performed at the most institutional end of the UK spectrum [11], highlighted that the layout could indicate a socio-spatial structure referring to what Goffman [12] calls total institutions.

The mental health checklist was developed and piloted in Europe and reflected the de-institutionalisation principles of that continent. It does not include local features that might be unique for a country. For instance, a feature that New Zealand mental health facilities share in common and that was not included within the checklist was that New Zealand facilities had a court room within every ward. This is used by people under community treatment orders as well as the service users in the ward. The court deals with people under the Mental Health Act, and so the location of these precludes the need to go to a criminal or district court within the public arena. The judge visits once a week for people who are waiting to review their status under the Act. All line up outside the courtroom, and space is a consideration as it is required for those waiting outside of the courtroom. Those waiting do not know the order that the judge will see them. As the holding of tribunals within wards is atypical and does not occur in many countries, the mental health checklist was not adapted to include this feature. Its inclusion within a country-specific modification would need to be based on a larger sample within the country.

Domesticity has been defined in Western terms. The institutional classification according to the mental health checklist could conflict with what might be considered domestic for the Māori culture. For example, single room accommodation might have a different meaning for Māori, who might prefer shared room accommodation, indicating that aspects such as privacy have a different connotation in different countries and cultures. Investigation through future research that extends the geographical areas might help to reveal those features that were of local versus global importance.

The research demonstrated that even though non-institutional environments are important in the design of the state psychiatric wards, there is potential for significant improvement. There were very few features that were universally present. Specifically, there was not a single facility without notices on circulation areas’ walls, which is a practice considered to be institutional. Effective communication might be achieved through alternative outlets other than walls. The presence of psychiatric offices and a clinic inside the ward is considered an essential component of the concept of a ward. Beyond this, there are many components that might be debated and open to discussion. This is of particular importance if we juxtapose it with the increased institutionalisation and anti-ligature of the wards. For example, the seclusion rooms that are present in every ward in New Zealand are not a universal feature of the psychiatric ward. In terms of domesticity, none of the wards had urinals, but apart from this, there was no other non-institutional feature that was widely accepted. The above findings lead to two very significant conclusions: (1) the fluidity of what exactly a psychiatric ward is from a built environment/equipment perspective and (2) the debatable nature to which institutional features are absolutely necessary in psychiatric wards and also the debatable nature of what non-institutional elements should be avoided in the name of any restriction, for example, infection control or anti ligature. It is important to note that for the most acute spectrum, we might look at the findings between England and New Zealand. There is potential for designers and mental health providers to be open to design possibilities. The checklist could be used not only as an evaluation tool but also as a tool of options to increase the non-institutional design factor of the psychiatric wards.

With greater and more extensive use, the training or creation a protocol for the use of the checklist, especially if the checklist were to be used by those other than built environment researchers, could be beneficial. The checklist was developed by an architect, and that influenced the terminology and language of the checklist. The clarifications that were required when implemented by those outside the architectural discipline demonstrated that adaptations or a clarifying protocol would be helpful if the checklist were to be used across disciplines. This might replace the need for architectural oversight, explanations and extend further use of the checklist as an independent tool that can be added to any methodology regarding psychiatric buildings.

The checklist has been used in three countries and across two continents. In terms of limitations, all countries were developed and might be considered to represent Western healthcare systems. Expanding the checklist usage and investigation to other countries, including those that are developing and especially those countries with distinctively different cultures, will help to inform the ability of the tool to reflect cultural sensitivities of what constitutes a non-institutional environment. This constitutes a significant opportunity for future research.

## Figures and Tables

**Table 1:** Frequency of domestic (residential) and institutional context and site features using data from facilities investigated in England, France and New Zealand combined (*n* = 16).

Context and Site Feature	R or I *	Percentage of Facilities with Feature (Number of Facilities with Feature)
Located in a residential area	R	63% (10)
Immediate neighbours including housing	R	38% (6)
Shops within walking distance within the neighbourhood	R	88% (14)
Public park or recreational area within walking distance	R	81% (13)
Paved pedestrian paths	R	75% (12)
Building façade similar to adjacent buildings	R	63% (10)
Windows of different sizes according to function	R	63% (10)
Used building materials such as stucco, brick and stone	R	75% (12)
Differentiated in terms of size from adjacent buildings	I	75% (12)
Different distances from the street compared with adjacent buildings	I	75% (12)
Different colour from nearby buildings	I	44% (7)
Different material selection from nearby buildings	I	44% (7)
Different unit parking from adjacent buildings	I	88% (14)
Existence of a label or inscription before or by the entrance	I	75% (12)
Did not have a designated waiting area outside the entrance	I	63% (10)

* R = Domestic (residential) feature; I = Institutional feature.

**Table 2:** Frequency of institutional features by mental health checklist category, country and facility.

	Percentage (Number of Institutional Features per Category/Total Number of Category Features)							
Country and Facility	All Features Included	Excludes Non-Applicable ^1^	All Features Included	Excludes Non-Applicable ^1^	All Features Included	Excludes Non-Applicable ^1^	All Features Included	Excludes Non-Applicable ^1^
	Context and Site Features		Building Features		Space and Room Features		All Category Features	
**England**								
1	32% (7/22)	32% (7/22)	30% (12/40)	23% (8/35)	24% (36/150)	22% (28/128)	26% (55/212)	23% (43/185)
2	73% (16/22)	73% (16/22)	64% (25/39)	66% (23/35)	47% (70/150)	45% (57/128)	53% (111/208)	52% (96/185)
3	45% (10/22)	45% (10/22)	63% (25/40)	63% (22/35)	43% (63/147)	43% (55/128)	47% (98/208)	47% (87/185)
4	64% (14/22)	64% (14/22)	68% (26/38)	69% (24/35)	40% (53/134)	39% (50/128)	48% (93/194)	48% (88/185)
5	41% (9/22)	41% (9/22)	49% (17/35)	49% (17/35)	43% (64/150)	41% (53/128)	43% (90/207)	43% (79/185)
6	64% (14/22)	64% (14/22)	67% (24/36)	66% (23/35)	52% (78/150)	48% (62/128)	56% (116/208)	54% (99/185)
7	73% (16/22)	73% (16/22)	67% (24/36)	66% (23/35)	59% (89/150)	58% (74/128)	62% (129/208)	61% (113/185)
All (1–7)	56% (86/154)	56% (86/154)	58% (153/264)	57% (140/245)	44% (453/1031)	42% (379/896)	48% (692/1445)	47% (605/1295)
**France**								
1	23% (5/22)	23% 5/22)	48% (19/40)	49% (17/35)	26% (39/150)	23% (30/128)	30% (63/212)	28% (52/185)
2	59% (13/22)	59% (13/22)	45% (18/40)	43% (15/35)	38% (56/147)	38% (48/128)	42% (87/209)	41% (76/185)
3	41% (9/22)	41% (9/22)	63% (25/40)	66% (23/35)	40% (58/146)	39% (50/128)	44% (92/208)	44% (82/185)
4	59% (13/22)	59% (13/22)	45% (18/40)	51% (18/35)	46% (68/147)	47% (60/128)	47% (99/209)	49% (91/185)
5	14% (3/22)	14% (3/22)	63% (25/40)	63% (22/35)	48% (70/147)	48% (61/128)	47% (98/209)	46% (86/185)
All (1–5)	39% (43/110)	39% (43/110)	53% (105/200)	54% (95/175)	39% (291/737)	39% (249/640)	42% (439/1047)	42% (387/925)
**New Zealand**								
1	73% (16/22)	73% (16/22)	65% (26/40)	74% (26/35)	58% (87/150)	60% (77/128)	61% (129/212)	64%(119/186)
2	86% (19/22)	86% (19/22)	89% (31/35)	89% (31/35)	51% (77/150)	55% (70/128)	61% (127/207)	65%(120/185)
3	36% (8/22)	36% (8/22)	55% (22/40)	63% (22/35)	53% (80/150)	63% (80/128)	52% (110/212)	59% (110/185)
4	91% (20/22)	91% (20/22)	75% (30/40)	71% (25/35)	62% (92/148)	63% (81/128)	68% (142/210)	68% (126/185)
All (1–4)	72% (63/88)	72% (63/88)	70% (109/155)	74% (104/140)	56% (336/598)	60% (308/512)	60% (508/841)	64% (475/740)

^1^ Excludes features that were non-applicable in one or more of the sixteen facilities investigated.

**Table 3:** Common domestic (residential) and institutional features across countries by the mental health checklist category.

	I or R ^1^	Common Features across Countries ^2^ (✓)			
		E and F	E and NZ	F and NZ	E, F and NZ
Total number of common mental health checklist features		9	18	15	6
**Common context and site features**					
Different distance between the facility and adjacent buildings	I	-	(✓)	-	-
Unit parking was different from adjacent buildings, for example, availability, size, location	I	-	(✓)	-	-
The front garden had different fencing and landscaping compared with adjacent gardens	I	-	(✓)	-	-
Number of common context and site features		0	3	0	0
**Common building features**					
Front door opened automatically or opened out	I	(✓)	(✓)	(✓)	(✓)
Front door was neither sliding or revolving	R	(✓)	-	-	-
There was not a closet or coat hanging area near the entrance	I	-	-	(✓)	-
There were no individual mailing boxes for service users directly accessible to the postman	I	(✓)	(✓)	(✓)	(✓)
There was an administration office near the entrance	I	-	-	(✓)	-
The length of corridors was long within autonomous wards/facilities	I	-	-	(✓)	-
Bathrooms did not open directly onto social areas and instead opened onto corridors or bedrooms	R	-	(✓)	-	-
Notices on circulation area walls or doors	I	(✓)	(✓)	(✓)	(✓)
Exit signs on circulation areas’ walls	I	-	-	(✓)	-
Outside areas were visible from interior social spaces	R	-	(✓)	-	-
Number of common building features		4	5	7	3
**Common space and room features**					
There was an administration office in the ward/facility	I	-	-	(✓)	-
There was more than one office inside the ward/facility	I	-	-	(✓)	-
There was a dedicated room for staff to retreat inside the ward/facility	I	-	-	(✓)	-
There were psychiatric offices attached or included	I	-	(✓)	(✓)	(✓)
There was not an office for a resident general practitioner	R	-	(✓)	-	-
An absence of a seclusion room	R	(✓)	-	-	-
There was a clinic in the ward/facility	I	(✓)	(✓)	(✓)	(✓)
There was a television in the lounge, or in one of the lounges or social areas	R	-	(✓)	-	-
The dining room was not in another building or unit	R	-	-	(✓)	-
There was not a walk-in cooler	R	-	(✓)	-	-
There were locked closets for food, or cleaning products etc	I	(✓)	-	-	-
There were locked storage areas	I	(✓)	-	-	-
There was one sink in the bathroom	R	-	(✓)	-	-
The bathroom had one toilet	R	-	(✓)	-	-
The bathroom had one shower	R	-	(✓)	-	-
The bathrooms did not have urinals	R	(✓)	(✓)	(✓)	(✓)
The bathroom did not have a hoist	R	-	-	(✓)	-
The WC in common areas did not have a self-cleaning toilet seat	R	-	(✓)	-	-
Number of common space and room features		5	10	8	3

^1^ Abbreviations: I = Institutional feature; R = Domestic (residential) feature. ^2^ Country abbreviations: E = England; F = France; NZ = New Zealand.

## Data Availability

Data sharing is not applicable to this article.

## References

[1] Wolfensberger W. (2011). Social Role Valorization: A Proposed New Term for the Principle of Normalization. Intellect. Dev. Disabil..

[2] Kandylis D. The Return from the Asylum of Leros in the Society of Larissa.

[3] Markus T. (1993). Buildings and Power: Freedom and Control in the Origin of Modern Building Types.

[4] Killaspy H. (2007). From the asylum to community care: Learning from experience. Br. Med. Bull..

[5] Chrysikou E. (2014). Architecture for Psychiatric Environments and Therapeutic Spaces.

[6] Robinson J.W., Emmon P., Graff M., Duerk D., Campbell D. (1984). A role for the architect in the environment- behavior research. The Challenge of Diversity.

[7] Chrysikou E., Spear S. (2021). Personal communication.

[8] HBN 35 Accommodation for People with Mental Illness. Part 3: Case Studies. https://www.thenbs.com/PublicationIndex/documents/details?Pub=NHS&DocID=247795.

[9] Seawright J., Gerring J. (2008). Case Selection Techniques in Case Study Research: A Menu of Qualitative and Quantitative Options. Polit. Res. Q..

[10] World Health Organisation. https://www.euro.who.int/en/health-topics/noncommunicable-diseases/mental-health/priority-areas/services-and-deinstitutionalization.

[11] Chrysikou E. (2019). Psychiatric institutions and the physical environment: Combining medical architecture methodologies and architectural morphology to increase our understanding. J. Healthc. Eng..

[12] Goffman E. (1961). Asylum.

